# Designing and analysing feasibility studies of complex interventions: challenges related to assessing stop/go criteria

**DOI:** 10.1186/1745-6215-14-S1-O20

**Published:** 2013-11-29

**Authors:** Michelle Collinson, Shamaila Anwar, Liz Graham, Kayleigh Burton, Tamar Pincus, David Owens, Amanda Farrin

**Affiliations:** 1 Leeds Institute of Clinical Trials Research, University of Leeds, Leeds, UK; 2 Leeds Institute of Health Sciences, University of Leeds, Leeds, UK; 3 Department of Psychology, University of London, Surrey, UK

## 

Randomised controlled trials (RCTs) are time-consuming and costly so funders often require evidence of feasibility before they will fund large scale trials^1^. Feasibility studies can provide invaluable evidence relating to the practicalities of conducting large RCTs and can improve their likelihood of success. However, conducting feasibility studies of complex interventions and deciding whether or not to proceed to a full RCT, is not always straightforward. We will present the challenges encountered during the design and analysis of two feasibility studies: OBI (Optimised Behavioural Intervention for avoidant chronic low back pain patients) and MIDSHIPS (Multicentre Intervention Designed for Self-Harm using Interpersonal Problem Solving) and discuss the steps taken to overcome them. Recruiting and treating participants in a limited number of centres, with few therapists, is a complex challenge for both of these feasibility studies and crucial to determining their success; we will present the lessons learnt from our experience. We will also discuss the impact of missing data on our ability to assess stop/go criteria with respect to proof-of-concept. Estimating follow-up questionnaire response rates is an important objective in both studies, hence we will discuss the methods employed to maximise data collection and present our approach for providing robust estimates of response rates for the phase III trials.
